# Maternal healthcare utilization in rural Bangladesh: A comparative analysis between high and low disaster-prone areas

**DOI:** 10.1371/journal.pgph.0001409

**Published:** 2023-07-31

**Authors:** Afroza Begum, Syed Abdul Hamid

**Affiliations:** 1 Department of Statistics, University of Chittagong, Chittagong, Bangladesh; 2 Institute of Health Economics, University of Dhaka, Dhaka, Bangladesh; University of Embu, KENYA

## Abstract

This study examined the disparity in antenatal care (ANC) visits and institutional delivery between high-disaster-prone (HDP) and low-disaster-prone (LDP) areas, defined based on multi-hazards, in Bangladesh and assessed the influencing factors using Andersen’s behavioral model. In this study, cross-sectional data of 345 mothers, who had live birth the year preceding the survey, were used from the second-round multipurpose survey of a longitudinal research project conducted in May-June 2011. Hierarchical multinomial logistic and binary logistic models were respectively used to assess the determinants of ANC contacts and choice of childbirth place. We found very low utilization of 4+ ANC visits in both HDP (20%) and LDP (15%) areas. The difference is also not significant. The strong influencing factors of receiving 4+ ANC were mother’s education, household size, income, and proximity to health facility. The level of institutional delivery was also low (21%), and no significant difference between HDP (15.2%) and LDP (25.7%) was found. However, in the case of institutional delivery, significant (*p-value* ≤ .01) difference was found in C-section between HDP (42%) and LDP (79%). A significant (*p-value* ≤ .05) difference was also found in the attendance of graduate doctors/gynecologists between HDP (58%) and LDP (88%). Mothers of HDP areas were 52 percent less likely to choose institutional delivery compared to those of LDP areas. Moreover, there was 30 percent less likelihood of choosing institutional delivery with an increase in distance to the nearest health facility. Specific demand-side (e.g., awareness raising, expanding maternal voucher scheme, covering more mothers under maternal allowance, and facilitating more income-generating activities especially off-farm ones) and supply-side interventions (e.g., providing training to local traditional birth attendants, and deployment of boat-based medical teams in coastal and *char* areas) need to be undertaken to increase institutional delivery, especially in HDP areas. However, the ultimate solution depends on adopting long-term measures to prepare facilities ready by filling the vacant posts and reducing absenteeism. Public-private partnerships modality can also be introduced especially in the HDP areas. Policy attention is needed to introduce such interventions.

## Introduction

Reducing the maternal mortality ratio (MMR) to 70 per 100,000 live births is a crucial indicator for achieving Sustainable Development Goals (SDGs) [1]. Another important indicator of SDGs is to reduce neonatal mortality to 12 or less per 1,000 live births [1]. However, MMR is still high in Bangladesh (173 deaths per 100 000 live births in 2017) despite its substantial decline over time (from 434 in 2000 to 173 in 2017) [2–4]. Although there is a sharp declining trend, the neonatal mortality rate also needs to be reduced from 19.1 to 12 per 1000 live births between 2019 and 2030 [1, 3]. The evidence shows that poor utilization of antenatal, delivery, and postnatal care services from qualified healthcare providers increases maternal, infant, and neonatal mortality [5–7]. Thus, further reduction in MMR requires strengthening the provision of maternal healthcare, especially antenatal care (ANC) and institutional delivery, which has been reflected in the Health, Nutrition, and Population Sector Program 2017–2022 of Bangladesh [8]. Evidence shows that Bangladesh is far behind in the utilization of four or more ANC visits (37%) as recommended by World Health Organization (WHO) [3]. Despite quite a progress, the rate of institutional delivery is still very low (47% in 2016) in Bangladesh [3, 5, 9, 10]. Thus, more attention needs to be given to increasing the WHO-recommended four or more ANC visits and institutional delivery in the left behind and climatically challenging areas such as disaster-prone areas for a further decline in maternal and neonatal mortality.

Plausibly there is a large variation in maternal healthcare utilization, especially in institutional delivery, among geographically diversified regions in the country, especially notably those experiencing different climate change risks, as livelihoods, natural calamities, healthcare infrastructure, etc., are not evenly distributed. The evidence in this regard is crucial for informed policy discussions. There is still a knowledge gap, as illustrated below, in the field of climatically, challenging areas despite a good number of studies on maternal healthcare use.

This study explores the variation in ANC visits and institutional delivery between the two broad geographical areas of Bangladesh: high- disaster-prone areas (HDP), where climate change risk is high, and low-disaster-prone areas (LDP), where it is not high. This can be generally expected that HDP areas have lower utilization of ANC and institutional delivery than the LDP areas. This study also assesses the influencing factors of these two outcomes using the conceptual framework of Andersen’s behavioral model (ABM) for healthcare utilization.

## Literature review

### ANC utilization

A growing body of studies in developing counties focused on utilization and determinants [11–17], and out-of-pocket payments [18] of ANC. Several studies also exist, as reviewed below, in the Bangladesh context [5, 9, 19–24].

A study using Bangladesh Demographic and Health Survey (BDHS) 2007 data found that about half of the respondents sought ANC from skilled providers [20]. This study also found mothers’ age and education, parity, area of residence, wealth index, desire for pregnancy, and wanting more children as the dominating factors of seeking ANC. Another study conducted in two sub-districts of Netrokona District revealed that about one-fourth of the pregnant women received four or more ANC while only 11 percent of women had their first ANC visit in the first trimester of pregnancy [20].

The common determinants of the utilization of ANC by a qualified provider in Bangladesh as evidenced in the literature were area of residence, educational level and employment status of mothers, husband’s occupation, and household’s access to mass media [5, 9, 20, 23, 24]. A number of factors such as the mother’s age at birth, education and occupation level of the mother and her spouse, parity, economic condition, the existing number of children, place of residence, media exposure, pregnancy intention status, and terminated pregnancy had an influence on four or more ANC visits [5, 22–24].

### Institutional delivery

Several studies also concentrated on the developing countries context in assessing the utilization [25–27], determinants [28–35], and inequalities [36] of institutional delivery. Some studies also exist in the Bangladesh context [37–49].

Huda et al. [37] explored the individual and community-related factors that influence institutional delivery in Bangladesh based on Bangladesh Maternal Mortality Survey 2010. Using BDHS 2011, another study found the prevalence of institutional childbirth and the related components in Bangladesh [38]. In 2006, another study assessed the socioeconomic determinants of seven areas of maternal and child health care, including child delivery care, using cross-sectional data on 128 purposively chosen remote villages in three divisions of Bangladesh [39]. To examine maternal complications, Biswas et al. [40] conducted a qualitative study using a focus group discussion with women with recent child delivery in a geographically challenging area of Bangladesh. Some studies examined the trends and/or inequities in ANC and institutional delivery in Bangladesh’s urban and rural areas using the BDHS or Bangladesh Maternal Mortality and Health Care Survey data [41–43]. Several studies assessed the influencing factors of child delivery care [44, 45, 47, 49]. Another study evaluated Bangladesh’s health system’s contribution to delivering adequate maternal healthcare for child delivery by trained birth attendants using the WHO framework for strengthening the health system [46]. A qualitative study used key informant interviews and focus group discussions to examine maternal healthcare utilization, including child delivery care, among adolescent girls in Bangladesh [48].

The evidence showed that the institutional delivery in Bangladesh is far below the average level of the developing countries [26, 29, 32]. Both individual and community-level factors influence institutional delivery in Bangladesh. The major community-level factors, as found in a study based on Bangladesh Maternal Mortality Survey 2010, were residential area, degree of poverty, media exposure, level of ANC use, and the number of educated women in the community [37]. The important individual-level factors were the mother’s age at birth, educational level of the mother and her husband, birth order, religion, complication in pregnancy, ANC use, area of residence, adoption of family planning methods, prior experience of institutional delivery, household’s access to mass media, socio-economic status of the household, and knowledge of available community clinic service [37–49]. Seeking four or more ANC visits was also found to be the influencing factor of the utilization of institutional delivery in a study that analyzed BDHS 2014 data by applying the propensity-score matching method [19].

The inequity in maternal healthcare utilization is also a concern in the Bangladesh context as documented in the literature. A number of studies concentrated on trends and/or equity in the utilization of ANC and delivery care [6, 9, 10, 50–52].

### Climate change risk and maternal healthcare use

Bangladesh, despite being a very small country, has a vast ecological diversity, e.g., plain land, riverbank and/or river island (*char*), water submerged (*haor*), and coastal areas. The latter three areas experience high climate change risks where the incidence of natural calamities (e.g., floods, cyclones, droughts, riverbank erosion, salinity, etc.) is higher. This ecological susceptibility leads to making the utilization of maternal healthcare more challenging in disaster-prone rural areas [53–57]. Thus, there is a plausibility of variation in maternal healthcare utilization across the regions experiencing different climate change risks. Research exploring the nexus between climate change risk and maternal healthcare use is crucial for policy discussion in Bangladesh due to its vulnerability to climate change and highly disaster-prone nature.

Most of the existing studies in the Bangladesh context measured socio-economic disparities [6, 9, 10, 50] where some analyzed the rural-urban comparisons and/or divisional comparisons [6, 22, 37, 51] of maternal healthcare use. However, few studies assessed the impact of climate change risks, such as flood and riverbank erosion on maternal healthcare use.

Using Bangladesh Demographic and Health Survey 2014 data and Emergency Events Database, one study assessed the effect of floods on maternal healthcare use [58]. This study found that flood, especially the recurrent flood, had a significant and negative impact on the utilization of maternal healthcare. Another study based on cross-sectional data from 25 disaster-prone villages in Bangladesh compared the pattern of receiving ANC between displaced, due to flood and/or riverbank erosion, and non-displaced households’ mothers [59]. This study found that the likelihood of seeking an ANC from a trained provider was lower among pregnant women from displaced households compared to those from non-displaced households. Using the same survey, another article found that mothers from displaced households were more likely to have institutional delivery than those from non-displaced households [60]. It was also evident that the level of institutional delivery decreased with an increase in prior displacement events. Moreover, maternal healthcare utilization (e.g., ANC, child delivery, and post-natal care) were significantly lower compared to the national average, as found in a study in *haor* areas [61]. Mothers’ age and education level were the leading factors of maternal healthcare use in these areas. A very recent study measured whether maternal healthcare utilization, both ANC and institutional delivery were affected by the natural disaster, floods [62]. Though this study found in bivariate analysis that floods have an impact on maternal healthcare usage, the multivariate analysis reveals the opposite conclusion.

The recall bias is a common issue of the former three studies due to considering a three to five years recall period, while the latter study is not nationally representative as it was conducted in only one district, Habiganj, of Bangladesh. Moreover, these studies were restricted to flood and/or riverbank erosion dimensions for defining the climate change risk. None of the studies included the *char*, *haor*, and coastal areas (which are highly vulnerable to climate change risks) combinedly. Hence there is a paucity of evidence on the disparity in the utilization of ANC and institutional delivery between the regions with high and low climate change risks defined based on multi-hazards (flood, riverbank erosion, and cyclone). However, such evidence has enormous importance *for priority setting* in any informed policy discussion. This gap in knowledge motivates this paper.

## Methodology

### Ethic statement

The study received ethical approval from the Institutional Review Board of the Institute of Health Economics, University of Dhaka, Dhaka. Verbal consent from the participants was solicited by informing them about the procedures and risks involved in the study. We sought verbal consent because most rural people usually want to refrain from participating in any survey giving written consent. The people of Bangladesh, especially those living in rural areas with low levels of education, have a common fear that they may lose ownership of their valuable properties by giving signatures on any document. The verbal consent was recorded in the questionnaire by asking whether they agreed to participate in the survey.

### Study design and setting

This study used cross-sectional data from the second-round survey of a longitudinal research project entitled “Microinsurance, Poverty and Vulnerability, which was housed at Inclusive Finance and Development (InM), Dhaka, Bangladesh, formerly known as the Institute of Microfinance (InM). This survey was conducted during May-June 2011. The survey used a multi-stage stratified sampling design under the Integrated Multipurpose Sample design framework.

In this survey, we included six *unions*, the smallest administrative unit in Bangladesh, from the first-round survey located in three districts (Mymensingh, Tangail, and Brahmanbaria) spread over two divisions of the country which comprises a sample of 2000 households. Capturing the geographical diversity along with making it nationally representative is the main reason for not including the full sample of the first-round survey conducted in 2009 over two divisions (details are illustrated in [63]. Additionally, 2200 households were included in second-round survey from 5 new randomly selected districts (Chapainawabganj, Chuadanga, Patuakhali, Sunamganj and Gaibandha) to capture all the seven divisions of the country and also keeping in view the geographical diversity (c*har*, *haor*, costal and boarder areas). One *upazila* (Nachole, Alamdanga, Bauphal, Jamalganj, and Palashbari) from each district and two *unions* for each *upazila* were selected randomly which yielded a total of ten *unions* from the 5 newly selected districts.

To match the sampling process of the first-round survey, the newly included *unions* were divided into ten strata (five with ‘in-patient’ care facilities and five without in-patient facilities). A sampling frame was formed by listing all the villages in each *union*. A sample of 7 villages was randomly selected from each *union* with an in-patient facility and 5 villages from each *union* without such a facility.

The sample size for each stratum was estimated using the following formula,

n=tα,N−12P1−Pd21+1Ntα,N−12P1−Pd2−1*Designeffect

with the population size *N* collected based on a sampling frame formed in April 2011, level of significance *α* = 0.05, margin of error *d* = 0.5 and design effect = 1.5. It is noted that, this survey was a multipurpose survey where the main outcome was ‘the proportion of individuals seeks healthcare for general illnesses. Thus, we selected this outcome as the parameter *P* where its value was considered as 0.3 based on the findings of the first-round survey.

Thus, using an equal allocation technique, the research team randomly selected 440 households from each of 5 newly selected districts yielding 2,200 (440*5) additional households in total. The total sample size in the second-round survey thus stood at 4,200 (2,000 + 2,200) households from 120 villages of 8 districts over 7 divisions. A total of 345 out of 3,791 households, which had one live birth over 12 months preceding the survey and were interviewed successfully, were the unit of analysis. We asked questions regarding household characteristics, including socio-economic factors, to the household heads since this survey was a multipurpose survey while maternal healthcare-related issues were asked to the mothers who gave childbirth over 12 months recall period to get more accurate data. In a few cases, the mothers, who gave childbirth over a 12 months recall period, were under 18 years of age. Although they had already become mothers, they were children by law. Thus, verbal consent was sought from their guardians (spouse/household head), and the interview was conducted in the presence of the spouse/household head.

We also conducted a Key Informant based village survey which covered details of physical, educational, and health infrastructures, literacy rate, the occurrence of macro shocks (floods, droughts, cyclones, river erosions, pest attacks, and so on), and the type of insurance products available locally. *Union Parisad* members, school teachers, religious leaders, and/or civil society leaders were considered as the respondents in the village survey.

### Data collection

We used a semi-structured questionnaire which was developed in the native language (Bengali). After an internal review, the questionnaire was sent to a group of experts for an external review. Incorporating the experts’ feedback, the revised questionnaire was made ready for the training of interviewers. After a thorough checking of inconsistencies and language suitability during training sessions, the questionnaire was geared up for testing. Incorporating the feedback received from the piloting process, the questionnaire was then finalized before being administered to the subjects. The survey was conducted via interviewers with 10 groups, each consisting of a field supervisor and four field investigators. The core research team visited all the survey areas to ensure the quality of the data collection process. In addition, a research assistant made unannounced field visits and verified the questionnaires from time to time. We prepared the data for analysis after necessary editing, coding, and consistency checking by the core research team.

A series of questions regarding antennal care (ANC) and delivery care and associated costs with coping mechanisms were posed to the respondents for the last childbirth over the 12 months preceding the survey. In addition, we collected information about demographic and socioeconomic conditions as well as healthcare-seeking behaviour for general ailments.

### Data analysis

#### Classification of disaster-prone areas

Topographically, riverbank and/or river islands (*Char*), water submerged (*Haor*), and coastal areas are considered as disaster-prone areas where climate change risk (like flood, cyclones, droughts, riverbank erosion, salinity etc.) is more prevalent. In our sample, Gaibandha, Sunamganj, and Patuakhali districts respectively belong to *char*, *haor*, *and* coastal areas. However, we classified the survey areas broadly into two categories such as high disaster-prone (HDP) and low disaster-prone (LDP) areas based on the multi-hazard score drawn from village survey data (Table 1). As such classification directly does not exist in the literature, following an existing study [64], we measured multi-hazard scores where we performed village-level analysis and then combined the scores to get the district-level scores.

**Table 1:** Multi-hazard score of sampled districts.

Type of area	Districts	Hazard Score			Multi-hazard score (Flood, Cyclone & Riverbank erosion)
		Flood	Cyclone	Riverbank erosion	
LDP	Mymensing	1	4.8	1	6.8
	Chuadanga	8.8	1	1	10.8
	Brahmanbaria	1	15.4	1	17.4
	Tangail	2.4	21.1	1	24.5
HDP	Giabandha	34.1	19.5	8.4	62
	Nawabganj	1	69.2	1	71.2
	Sunamganj	73.7	1	1	75.7
	Patualhali	106	107.2	58.5	271.7

The village survey captured the incidences of various disasters including flood, riverbank erosion and cyclone, with the level of perceived severity (severe, moderately severe, and not severe) during the last five years preceding the survey. The multi-hazard score obtained from the village survey data ranged from 6.8 to 271.7. We considered the districts that scored ≤ 50 were considered as LDP, and those that scored > 50 as HDP areas. In this consideration, Mymensing, Chuadanga, Brahmanbaria and Tangail fell into LDP areas and Gaibandha, Nawabganj, Sunamganj and Patuakhali fell into HDP areas. As mentioned earlier, Gaibandha, Sunamganj and Patuakhali districts belong to *char*, *haor and* coastal areas respectively where climate change risks are higher; thus, this clearly implies that the classification of HDP and LDP areas are well justified.

#### Outcome variables

We used two outcome variables. One outcome was the number of ANC contacts which were categorized as none (no visit), inadequate (1–3 visits), and adequate or recommended (four or more visits) according to previous guidelines of WHO. It is noted that WHO now-a-days recommends eight or more ANC visits between conception and childbirth [65], which is not feasible in the rural context of Bangladesh [19, 21]. The choice of the place for giving childbirth, as the second outcome variable, was categorized as institutional setting and at home.

#### Selection of predictors

**Fig 1:**
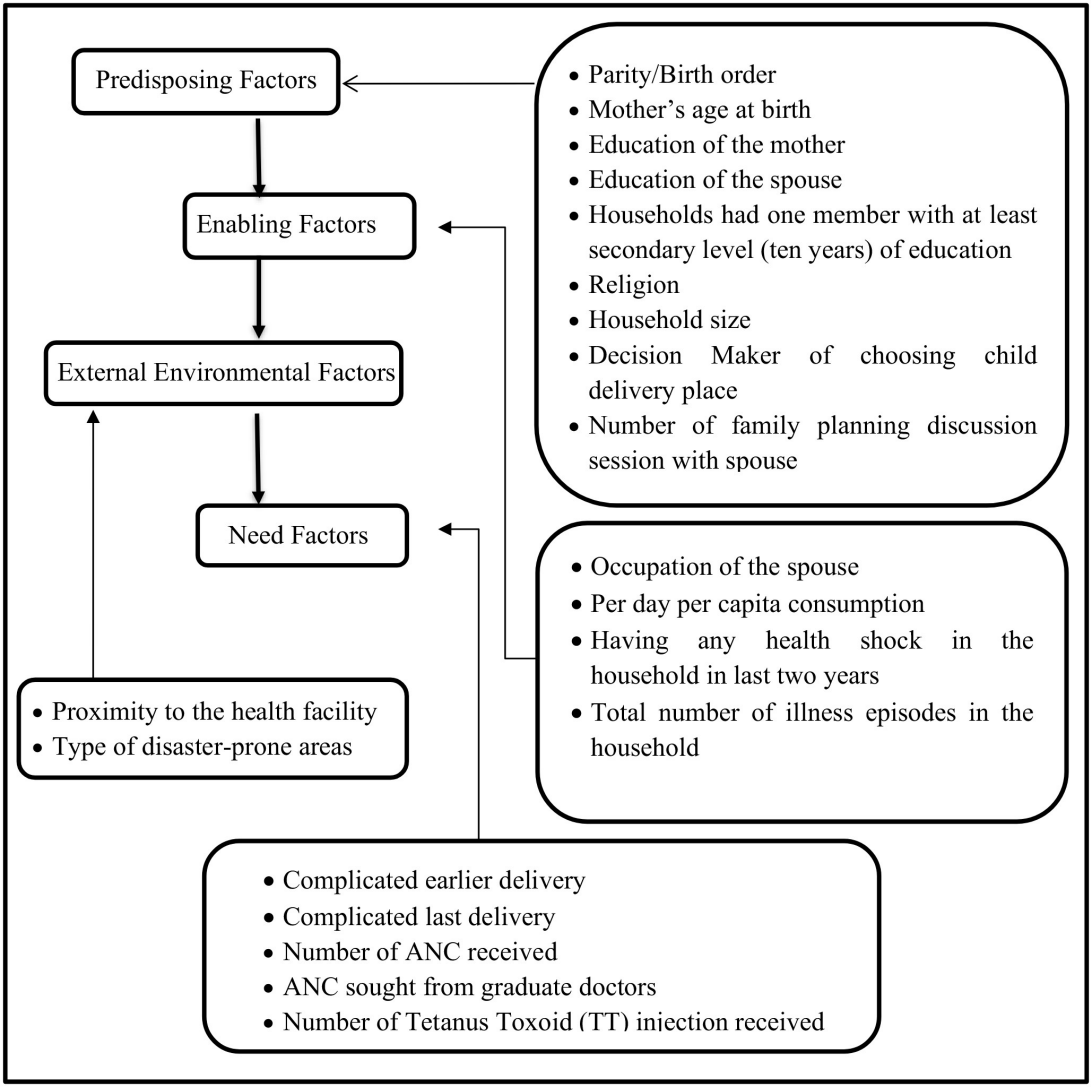
List of variables according to the conceptual framework of Andersen Behaviour model.

Healthcare use is influenced by both demand-side and supply-side factors. As mentioned earlier, the existing literature identified the wide-ranging dominating factors of maternal healthcare use (ANC use and institutional delivery). However, the influential factors of these outcomes differ over socio-economic and demographic perspectives, various cultures, and ecological contexts [16, 39, 66]. We applied the Andersen Behaviour model [67] for examining these factors systematically, as used in some earlier studies [14–16, 22, 32, 35, 39]. This model hypothesizes that health service use is influenced by predisposing, enabling, external environmental, and need factors, as illustrated in Fig 1. Predisposing factors refer to the socio-demographic features, and enabling factors infer the affordability of accessing the services. The external environment factors reveal the availability to access the services and the characteristics of the healthcare delivery system. The need factors enable the perceived health status and the illness conditions which directly influence the utilization of healthcare [67].

However, some variables such as the decision maker of choosing the child delivery place, number of ANC received, ANC sought from graduate doctors, and number of TT received were relevant for the second outcome (choice of the place for giving childbirth) only. Thus, these variables were only used as potential factors of choice of child delivery place.

#### Statistical methods

We used both bivariate and multivariate analyses. In the bivariate analysis, we simply compared background characteristics, utilization of ANC, and institutional delivery between HDP and LDP areas. Z-tests (t-tests) were performed to assess the robustness of the differences in the proportion (mean) of the categorical (numerical) variables between HDP and LDP areas. It is noted that we, following the earlier studies [68–70], classified the health care use (ANC or delivery) as formal care if that was received from any qualified medical care providers (e.g., MBBS doctor, gynecologist/surgeon, medical assistant, nurse, paramedic, and government and NGO community health workers). The delivery care provided by a trained traditional birth attendant (TTBA) and skilled birth attendant (SBA) was also considered as formal care. The remaining categories of healthcare providers (quack, homeopathic healers, traditional healers, TBA, etc.) were defined as informal providers.

In multivariate analysis, hierarchical multinomial logistic regression was used to assess the factors of ANC contacts. In addition, the hierarchical binary logistic model was used to examine the determinants of the choice of childbirth place. Note that the gradual inclusion of explanatory variables with equal sample size in each model of the hierarchical regression analysis leads to estimating the best-fitted model from the empirical data. Multicollinearity among the independent variables was tested with variance inflation factor (VIF), while the result (tolerance <1 and VIF < 2) indicates no multi-collinearity [71]. Thus, the predisposing, enabling, external environmental, and need factors, as mentioned above, were regressed individually against each outcome variable. According to the prior studies [11, 72, 73], the predictors, which were significant at 25 percent level or below in the unadjusted regression model, were included in the adjusted hierarchical model of both utilization of ANC and choice of childbirth place. Since there were four groups of potential factors-, we used the hierarchical regression for examining the effect of each group of factors distinctly as well as controlling and adjusting the interactions between the different groups [32].

In the adjusted hierarchical model, we ran the regression on the predisposing factors in Model I. In Model II, the regression was run on enabling factors, and the factors of Model I obtained significant at 15 percent level or below following the literature [32, 74].

In Model III, the regression was run on external environmental factors, and the factors of Model II obtained significant at 15 percent level or below. In Model IV, the final regression was run based on the need factors, and the variables of Model III found significant at 15 percent level or below. We used Hosmer–Lemeshow goodness-of-fit test [75] to check whether the model fits well. We excluded two observations from the regression due to missing data. All the analyses were performed using STATA 13.

## Findings

### Background characteristics

A total of 345 out of 3,791 (1,605 from HDP areas and 2,186 from LDP areas) successfully interviewed households reported a single live birth over 12 months preceding the survey, of which 46% were in HDP areas and 54% in LDP areas. The reported pregnancy was second or third for half of the cases.

Table 2 shows the descriptive statistics of individual-level and household-level characteristics of the mothers. The mothers were, on average, 26 years old and their ages ranged from 16 to 45. The mothers had, on average, 5 years of schooling while their spouse had 4 years of schooling. About 49 percent of spouses had employment in the agriculture sector, followed by business (17%) and service (13%). The average household size was 6, and per day per capita consumption, a proxy measure of income, was BDT 53 (below 1 USD). The average distance from the homestead to the nearest health facilities was 1.27 miles. There is a statistically significant difference between HDP and LDP areas for some factors such as the educational level of mothers (*p-value* ≤ .10) and their spouse (*p-value* ≤ .05), per capita consumption (p-value ≤ .01), and proximity to the nearest health facility (p-value ≤ .01). Statistically significance difference is also seen for an occupational category (i.e., agriculture) of the spouse.

**Table 2:** Background characteristics and reproductive status of the study participants.

Variables	Categories	HDP $\left(\begin{array}{c} \%\\ N \end{array}\right)$ *or* (*Mean* ± *SD*)	LDP $\left(\begin{array}{c} \%\\ N \end{array}\right)$ *or* (*Mean* ± *SD*)	*p*-value^1^	Total $\left(\begin{array}{c} \%\\ N \end{array}\right)$ *or* (*Mean* ± *SD*)
**Predisposing factors**					
Parity/birth order	First	34.81 (55)	33.16 (62)	0.85	33.91 (117)
	Second or third	44.94 (71)	54.55 (102)	0.21	50.14 (173)
	Fourth or higher	20.25 (32)	12.30 (23)	0.82	15.94 (55)
Age of the mother (in years)		25.73±5.58	25.55±5.78	0.77	25.63±5.68
Education of the mother (in years)		4.36±3.52	5.36±3.58	0.06	5.03±3.57
Religion	Muslim	94.94 (150)	96.26 (180)	0.56	95.65 (330)
	Others	5.06 (8)	3.74 (7)	0.90	4.35 (15)
Education of spouse (in years)		3.72±3.89	4.76±4.14	0.02	4.28±4.06
Households had one member with at least secondary level (ten years) of education	Yes	18.35 (29)	30.48 (57)	0.23	24.93 (86)
Household size		5.99±2.65	5.85±1.99	0.57	5.92±2.31
Number of family planning discussion session with spouse	Multiple times	54.43 (86)	63.10 (118)	0.21	59.13 (204)
	Otherwise	45.57 (72)	36.90 (69)	0.30	40.87 (141)
**Enabling Factors**					
Occupation of spouse	Agricultural sector	62.42 (98)	37.97 (71)	0.00	49.13 (169)
	Transport sector	7.64 (12)	11.76 (22)	0.71	9.88 (34)
	Business sector	12.1 (19)	20.86 (39)	0.42	16.86 (58)
	Service sector	10.19 (16)	16.04 (30)	0.59	13.37 (46)
	Other	7.64 (12)	13.37 (25)	0.61	10.76 (37)
Per day per capita consumption (a proxy measure of income)		57.25±32.96; Median = 48.11	70.63±52.79; Median = 56.94	0.01	64.51±45.25; Median = 52.79
Having any health shock in the household in last two years	Yes	37.97 (60)	36.36 (68)	0.85	37.10 (128)
Total number of illness episodes for all members in the household over 12 months period		2.54±1.47	2.55±1.19	0.99	2.54±1.32
**External Environmental Factors**					
Distance to the nearest health facility (in mile)		1.65±1.88	0.96±0.98	0.00	1.27±1.50
**Need Factors**					
Having any complication in the last delivery	Yes	20.89 (33)	18.18 (34)	0.78	19.42 (67)
Having any complication during the earlier gestation	Yes	9.49 (15)	11.76 (22)	0.83	10.72 (37)

^1^. Z-tests were performed to assess robustness of the differences in the proportion of the categorical variables while t-tests were used to check the robustness of the differences in mean of the numerical variables between HDP and LDP areas.

About 19% (67 out of 345 cases) of the mothers had some complications during their last pregnancy, where excessive weakness (24%) and abdominal pain (19%) were the main problems. About 11% of the mothers also had complications in their earlier pregnancies, of which the majority (54%) had some complications during the last pregnancy. The difference is not statistically significant between HDP and LDP areas in each state. We used ‘the number of family planning discussion sessions with the husband’ as a proxy variable for an intimate relationship with the husband, which potentially affects the utilization of ANC and delivery care. The result shows that about three fourth of pregnant women made any discussion session on family planning with their spouse (59% discussed more than once and 16% discussed only once) while the remaining 25% never made any discussion session.

### Antenatal care

About 67% of women reported that they sought some ANC (Table 3). However, the difference between HDP (63%) and LDP (71%) areas is not significant in the utilization of ANC. ANC utilization from formal healthcare providers was a bit higher in LDP areas (95%) than in HDP (93%). ANC visits to graduate doctors were also somewhat higher in LDP (55%) compared to HDP (52%). However, the difference is not significant in any case.

**Table 3:** Pattern of antenatal care contacts.

Particulars	Categories	HDP areas % (n)	LDP areas % (n)	*p-value* ^1^	Total % (n)
Sought any ANC		63.29 (100)	70.59 (132)	0.24	67.25 (232)
Type of ANC	Formal care	93.00 (93)	95.45 (126)	0.44	94.40 (219)
	Informal care	7.00 (7)	4.55 (6)	0.85	5.60 (13)
Provider of ANC	Graduate doctor	52.00 (52)	54.54 (72)	0.78	53.45 (124)
	Others	48.00 (48)	45.46 (60)	0.79	46.55 (108)
No. of ANC visits	None (no visit)	36.71 (58)	29.41 (55)	0.41	32.75 (113)
	Inadequate (1–3 visits)	43.04 (68)	55.61 (104)	0.10	49.86 (172)
	Recommended (≥4 visits)	20.25 (32)	14.97 (28)	0.59	17.39 (60)
Reasons for not seeking any ANC	Not needed	48.28 (28)	69.09 (38)	0.09	58.41 (66)
	Inability to pay	27.59 (16)	23.64 (13)	0.81	25.66 (29)
	Proximity to healthcare providers	6.90 (4)	1.82 (1)	0.85	4.42 (5)
	Others	17.23 (10)	5.45 (3)	0.61	11.5 (13)
No. of TT injection received	None	27.22 (43)	18.18 (34)	0.35	22.32 (77)
	1–4 doses	58.86 (93)	79.15 (148)	0.00	69.85 (241)
	5 doses	13.92 (22)	2.67 (5)	0.48	7.83 (27)
Ultrasonography	yes	27.85 (44)	51.34 (96)	0.01	40.58 (140)

^1^. Z-tests were used to assess the differences in the proportion of the categorical variables between HDP and LDP areas.

About 17 percent of the mothers sought a minimum of four ANC visits as recommended in the previous guideline of WHO, while only 1.45 percent of women received eight or more ANC visits as recommended in the new guideline. Note that the differences among the different groups of ANC visits categorized as none (no visit), inadequate (1–3 visits), and recommended (≥4 visits) are significantly significant (*p-value* ≤ .05). However, there is no statistically significant difference between HDP (20%) and LDP (15%) areas in the utilization of recommended ANC. The utilization of inadequate ANC (1–3 visits) was higher, but not significant at 5 percent level, in LDP areas (56%) than in HDP areas (43%). About 26 percent of those who did not seek ANC reported inability to pay as a reason for not seeking ANC, while proximity to healthcare providers was reported by 4 percent of pregnant women. About 78 percent of mothers received at least one dose of TT vaccine, while about 70 received 1–4 doses, and about 8 percent received 5 doses. However, the difference between HDP (73%) and LDP (82%) areas is not significant at the 5 percent level. Ultrasound scanning was used by about 41 percent of pregnant women, which is significantly (*p-value* ≤ .01) lower in HDP (28%) than in LDP (51%) areas.

We initially ran unadjusted regression models to identify the predictors of ANC visits for using them in the adjusted hierarchical regression model as mentioned in the ‘Statistical Methods’ section. The unadjusted results show that parity/birth order, educational level of the pregnant woman and her spouse, religion, household size, number of family planning discussion sessions with the spouse, spouse’s occupation, household income, number of illness episodes in the household, proximity to the health facility, type of disaster-prone areas, complications in earlier pregnancy and complications in the last pregnancy were significantly (p-value ≤ 0.25) associated with utilization of ANC visits (S1 Table).

Table 4 presents the relative risk ratio of the influencing factors of ANC visits estimated using the multinomial logistic regression. Model I of adjusted hierarchical regression reveals that all the predisposing factors apart from parity had a significant relationship at a 15 percent level with ANC contacts. In Model II, all the significant predisposing factors of Model I and some newly included factors, such as household income, and the total number of illness episodes in the household, were significant at the 15 percent level. All the significant predisposing and enabling factors of Model II and the external environmental factors such as proximity to health facilities and type of disaster-prone areas added in Model III were found significant at the 15 percent level.

**Table 4:** Multinomial logistic model estimation to identify the determinants of ANC contacts.

Explanatory variables	*Dependent variable*: Number of ANC received (None (0) is the reference category)							
	Adjusted model							
	Model I		Model II		Model III		Model IV	
	1–3	≥ 4	1–3	≥ 4	1–3	≥ 4	1–3	≥ 4
	RRR (CI)	RRR (CI)	RRR (CI)	RRR (CI)	RRR (CI)	RRR (CI)	RRR (CI)	RRR (CI)
**Predisposing factors**								
Parity								
First	Ref	Ref						
Second or third	0.78 (0.42 to 1.43)	0.97 (0.45 to 2.09)						
Four or more	1.05 (0.47 to 2.33)	0.51 (0.12 to 2.09)						
Mother’s Education (in years)	1.07^§^ (0.98 to 1.18)	1.21*** (1.06 to 1.38)	1.08* (0.98 to 1.19)	1.27*** (1.11 to 1.45)	1.07^§^ (0.98 to 1.18)	1.25*** (1.10 to 1.43)	1.07 (0.98 to 1.18)	1.25*** (1.10 to 1.43)
Religion (1 = Muslim)	0.12** (0.01 to 1.03)	0.11* (0.01 to 1.14)	0.12** (0.01 to 1.02)	0.10* (0.01 to 1.11)	0.15* (0.02 to 1.22)	0.14 (0.01 to 1.51)	0.16* (0.02 to 1.37)	0.15 (0.01 to 1.56)
Spouse’s Education (in years)	1.13*** (1.04 to 1.23)	1.12** (1.00 to 1.24)	1.10** (1.01 to 1.22)	1.09^§^ (0.97 to 1.22)	1.11** (1.02 to 1.21)	1.09 (0.98 to 1.22)	1.11** (1.02 to 1.21)	1.09* (0.98 to 1.22)
Household size	0.84*** (0.75 to 0.94)	0.81*** (0.69 to 0.95)	0.84*** (0.75 to 0.95)	0.78*** (0.66 to 0.92)	0.85*** (0.75 to 0.95)	0.80*** (0.68 to 0.94)	0.85*** (0.75 to 0.95)	0.80*** (0.68 to 0.94)
Number of family planning discussion session with spouse (1 = Multiple times, 0 = otherwise)	1.90** (1.13 to 3.21)	2.27** (1.10 to 4.69)	1.77** (1.05 to 2.98)	2.03* (0.97 to 4.23)	1.73** (1.02 to 2.91)	2.03 (0.97 to 4.26)	1.65* (0.97 to 2.80)	1.89* (0.89 to 3.99)
**Enabling Factors**								
Occupation of spouse								
Agricultural sector	Ref	Ref	Ref	Ref				
Transport sector dummy			1.26 (0.52 to 3.04)	1.26 (0.52 to 3.04)				
Occupation of Spouse								
Business sector dummy			0.67 (0.31 to 1.42)	0.67 (0.31 to 1.42)				
Service sector dummy			1.22 (0.46 to 3.26)	1.22 (0.46 to 3.26)				
Other dummy			1.50 (0.61 to 3.73)	1.50 (0.61 to 3.73)				
Per day per capita consumption (log value)			1.77* (0.92 to 3.40)	1.77* (0.92 to 3.40)	1.59 (0.84 to 3.04)	1.98* (0.89 to 4.37)	1.58 (0.83 to 3.02)	1.98* (0.89 to 4.44)
Total number of illness episodes for all members in the household			.16 (0.94 to 1.43)	1.38** (1.06 to 1.81)	1.12 (0.91 to 1.39)	1.32** (1.02 to 1.72)	1.13 (0.91 to 1.39)	1.32** (1.02 to 1.72)
**External Environmental factors**								
Proximity to health facility (in miles)					0.88^§^ (0.73 to 1.05)	0.73** (0.54 to 0.98)	0.86* (0.71 to 1.03)	0.70** (0.51 to 0.95)
Type of disaster-prone areas (1 = HDP areas)					0.87 (0.50 to 1.49)	1.94* (0.94 to 4.03)	0.88 (0.51to 1.52)	2.01* (0.97 to 4.21)
**Need Factors**								
Complicated earlier delivery (1 = yes)							1.88* (0.89to 3.96)	1.73 (0.66 to 4.56)
Complicated last delivery (1 = yes)							1.81 (0.68to 4.80)	2.67 (0.78 to 9.17)
Number of observations	343		343		343		343	
Hosmer–Lemeshow goodness-of-fit test	Number of groups = 10 chi-squared statistic = 5.348 degrees of freedom = 16 Prob > chi-squared = 0.994							

*Note*s:

***, **, *, ^§^ and ^¶^ indicates significance at 1%, 5%, 10%, 15%, 25% level, respectively. Ref = Reference Category; RRR = Relative Risk Ratio, CI = confidence interval, ANC = antenatal care.

Along with the need factors, all predisposing, enabling, and external environmental factors found significant at the 15 percent level in Model III were included in Model IV to run the final regression model of ANC contacts. This model fits well as per the Hosmer–Lemeshow goodness-of-fit test. The results of the final model show that inadequate ANC visits (1–3), relative to no ANC visit, were more likely to receive by the pregnant women who had spouses with more years of schooling (RRR: 1.11; *p-value ≤ 0*.*05*), who made more than one-time family planning discussion session with her spouse (RRR: 1.65; *p-value ≤ 0*.*10*) and who had complications in earlier pregnancies (RRR: 1.88; *p-value ≤ 0*.*10*).

The likelihood of receiving 1–3 ANC contacts, relative to no care, (RRR: 0.16; *p-value ≤ 0*.*10*) was lower among Muslim women compared to non-Muslim women. Similarly, women with more family members were less likely to seek 1–3 ANC contacts (RRR: 0.85; *p-value ≤ 0*.*01*) relative to no care. Women with more family members were also less likely to seek recommended ANC (≥ 4) (RRR: 0.80; *p-value ≤ 0*.*01*) relative to no care.

However, mothers with more years of schooling (RRR: 1.25; *p-value ≤ 0*.*01*) were 25 percent more likely to seek recommended ANC (≥ 4) relative to no care. The likelihood of receiving four or more ANC decreased with increased household size (RRR: 0.80; *p-value ≤ 0*.*01*) and increased with augmented household income (RRR: 1.98; *p-value ≤ 0*.*10*). Surprisingly, a number of illness episodes in the household (RRR:1.32; *p-value* ≤ 0.05) were positively associated with the recommended number of ANC (≥ 4) visits. Proximity to health facilities was an important factor in using the recommended ANC contacts. The likelihood of using the recommended ANC decreased with increasing the distance to the nearest health facility (RRR: 0.70; *p-value ≤ 0*.*05*). Unexpectedly, the likelihood of using the recommended number of ANC was higher (RRR: 2.01; *p-value ≤ 0*.*10*) among the women living in HDP areas compared to those in LDP areas.

### Delivery care

Table 5 shows that 79 percent of childbirths occurred at home whereas only 21 percent at the institutions of which 67 percent was C-Section delivery. LDP areas (79%) had significantly (*p-value* ≤ .01) higher C-section delivery compared to HDP areas (42%). The incidence of home delivery was significantly (*p-value* ≤ .01) higher in HDP (84.8%) than in LDP (74.3%) areas. On the other hand, the incidence of institutional birth was 41 percent lower, but not significant at any conventional level, in HDP (15.2%) than in LDP (25.7%). In the case of home deliveries, skilled attendants, especially TTBA/SBA, were present for 44 percent of cases. The presence of skilled attendants was considerably lower in HDP than in LDP areas. For institutional deliveries, the presence of graduate doctors/gynecologists was significantly (*p-value* ≤ .05) lower in HDP areas (58.3%) compared to the LDP areas (87.5%).

**Table 5:** Pattern of child delivery care.

Categories			HDP areas % (n)	LDP areas % (n)	*p-value* ^1^	Total % (n)
**Distribution of healthcare providers in delivery place**	**At home**		84.81 (134)	74.33 (139)	0.03	79.13 (273)
	Skilled attendants	by TTBA/SBA	26.12 (35)	41.73 (58)	0.13	34.07 (93)
		by Paramedic/FWV or NGO health worker	8.21 (11)	2.16 (3)	0.71	5.13 (14)
		by graduate doctor/nurse/gynecologist/Medical assistant	3.73 (5)	5.04 (7)	0.91	4.39 (12)
		Total	38.06 (51)	48.93 (68)	0.24	43.59 (119)
	Unskilled attendants	by TBA/quack	35.07 (47)	31.65 (44)	0.73	33.33 (91)
		by others	26.87 (36)	19.42 (27)	0.49	23.08 (63)
		Total	61.94 (83)	51.07 (71)	0.17	56.41 (154)
	**At institution**		15.19 (24)	25.67 (48)	0.31	20.87 (72)
		**Type of Physicians**				
		by graduate doctor/gynecologist/surgeon	58.33 (14)	87.50 (42)	0.02	77.78 (56)
		by others	41.67 (6)	12.50 (10)	0.18	22.22 (16)
		**Type of Providers**				
		Government	45.83 (11)	20.83 (10)	0.23	29.17 (21)
		Private	50.00 (12)	77.08 (37)	0.07	68.06 (49)
		NGO	4.17 (1)	2.08 (1)	-	2.78 (2)
		**Type of delivery**				
		Normal	50.00 (12)	20.83 (10)	0.16	30.56 (22)
		Assisted	8.33 (2)	0 (0)	-	2.78 (2)
		C-section	41.67 (10)	79.17 (38)	0.00	66.67 (48)
**Decision maker of selecting child delivery place**		Household head	15.82 (25)	19.79 (37)	0.70	17.97 (62)
		Husband	16.46 (26)	21.93 (41)	0.58	19.42 (67)
		husband-wife both	48.73 (77)	40.64 (76)	0.31	44.35 (153)
		Others	18.99 (30)	17.64 (33)	0.89	18.27 (63)
		Average of Out-of-pocket payments of maternal healthcare (both ANC and Delivery care) (in BDT)	2,378 [3,573] (158)	5,065 [8,133] (187)	0.00	3,835 [6,587] (345)

^1^. Z-tests were performed to assess the robustness of the differences in the proportion of the categorical variables, while t-tests were used to check the robustness of the differences in the mean of the numerical variables between HDP and LDP areas.

It is also seen that about 77 percent of mothers in LDP areas who sought institutional delivery visited private facilities, which was only 50 percent in HDP areas. The difference is significant at the 10 percent level. It is worth mentioning that 80 percent of cases who sought institutional delivery from private healthcare facilities had C-section delivery. This implies a strong correlation between C-section delivery and visits to private hospitals for institutional delivery. The childbirth place was selected jointly by husband-wife for about 44 percent of cases and by the husband alone for 19% of cases. However, there is no significant difference between HDP and LDP areas in this regard. It is noted that HDP areas had significantly (*p-value* ≤ .01) lower out-of-pocket payments for maternal healthcare (both ANC and delivery) compared to LDP areas.

The results of the unadjusted regression model reveal that all the potential factors, as mentioned earlier, except household size and households having any member with a secondary level or more education were associated with institutional delivery at the 25 percent significant level (S2 Table). Table 6 displays the adjusted logistic regression run for examining the determinants of choosing a child delivery place. In Model I, all the predisposing factors except the household size were associated with the choice of delivery place at the 15 percent significant level. In Model II, some predisposing and enabling factors such as parity, education and occupation of the spouse, and number of family planning discussion sessions with a spouse were significantly associated at 15 percent level with a choice of delivery place. The significantly associated predisposing and enabling factors of Model II and external environmental factors such as distance to the nearest health facility (in miles) and type of disaster-prone areas added in Model III were significantly associated at 15 percent level with a choice of delivery place.

**Table 6:** Logistic model estimation to assess the factors of choice of child delivery place.

Explanatory variables	*Dependent variable*: *choice of child delivery place (1 = at institution; 0 = at home)*			
	Adjusted model			
	Model I	Model II	Model III	Model IV
	OR (CI)	OR (CI)	OR (CI)	OR (CI)
**Predisposing factors**				
Parity				
First	Ref	Ref	Ref	Ref
Second or third	0.51* (.24 to 1.05)	0.48** (.26 to 0.90)	0.46** (.25 to 0.86)	0.30** (.13 to 0.62)
Four or more	0.11** (.02 to .71)	0.11*** (.02 to .51)	0.10*** (.02 to .43)	0.06*** (.008 to .38)
Mother’s age at birth (in years)	1.02 (.94 to 1.11)	-	-	-
Mother’s Education (in years)	1.09^§^ (.97 to 1.22)	1.05 (.97 to 1.22)	-	-
Religion (1 = Muslim)	0.56 (0.17 to 1.88)	-	-	-
Spouse’s Education (in years)	1.15*** (1.05 to 1.26)	1.14*** (1.04 to 1.26)	1.18*** (1.09 to 1.28)	1.16*** (1.06 to 1.28)
Decision maker of choosing child/s birthplace (1 = Husband-Wife together; 0 = Others)	0.92 (0.51 to 1.64)	-	-	-
Number of family planning discussion sessions with spouse (1 = Multiple times, 0 = otherwise)	1.78* (0.94 to 3.39)	2.04** (1.06 to 3.94)	1.99** (1.04 to 3.82)	1.74 (0.84 to 3.61)
**Enabling Factors**				
Occupation of Spouse				
Agricultural sector	Ref	Ref	Ref	Ref
Transport sector dummy		1.20 (.42 to 3.44)	0.98 (.34 to 2.80)	0.96 (.27 to 3.39)
Business sector dummy		2.01* (.88 to 4.59)	2.02* (.89 to 4.54)	2.79** (1.11 to 7.03)
Service sector dummy		1.20 (.50 to 2.91)	1.08 (.44 to 2.59)	0.99 (.36 to 2.73)
Other dummy		0.40^§^ (.12 to 1.37)	0.32* (.10 to 1.22)	0.13*** (.03 to 0.63)
Per day per capita consumption (log value)		1.45 (0.78 to 2.70)	-	-
Having any health shock in the household in the last two years (1 = yes; 0 = No)		0.71 (0.37 to 1.35)	-	-
Total number of illness episodes for all members in the household		0.87 (0.68 to 1.11)	-	-
**External Environmental factors**				
Proximity to health facility (in miles)			0.79^§^ (.58 to 1.06)	0.70* (.48 to 1.01)
Type of disaster-prone areas (1 = HDP areas)			0.64^§^ (.35 to 1.20)	0.48** (.22 to 1.01)
**Need Factors**				
Complicated earlier delivery (1 = yes)				3.87** (1.28 to 11.70)
Complicated last delivery (1 = yes)				4.67*** (2.05 to 10.65)
The number of ANC received				
None	Ref	Ref	Ref	Ref
Inadequate (1–3 visits)				1.57 (0.50 to 5.04)
Recommended (4 or more visits)				3.08* (0.89 to 10.6)
ANC sought from graduate doctors (1 = Yes; 0 = No)				3.49*** (1.58 to 7.68)
No. of TT received				
None	Ref	Ref	Ref	Ref
Inadequate (1–4)				1.20 (.44 to 3.31)
Adequate (5)				2.48 (0.58 to 10.65)
Number of observations	343	343	343	343
Hosmer–Lemeshow goodness-of-fit test	Number of groups = 10 Hosmer-Lemeshow chi2(8) = 10.15 Prob > chi2 = 0.2546			

*Note*s:

***, **, *, ^§^ and ^¶^ indicates significance at 1%, 5%, 10%, 15%, 25% level, respectively. Ref = Reference Category; OR = Odds Ratio, CI = confidence interval, ANC = antenatal care.

Model IV contains all predisposing, enabling, and external environmental factors found significant at the 15 percent level in Model III, along with the need factors in order to fit the final model of choice of child delivery place. According to Hosmer–Lemeshow goodness-of-fit test, this model also fits well.

In the final model, there was less likelihood of choosing institutional delivery for the mothers who had higher order of pregnancies (OR: 0.30; 0.06; *p-value ≤ 0*.*05*), had more distance to the nearest health facility (OR: 0.70; *p-value ≤ 0*.*10*) and were the inhabitants of HDP areas (OR: 0.48; *p-value ≤ 0*.*05*). The likelihood of choosing institutional delivery decreased by 30 percent with increased distance to the nearest health facility. Moreover, women in HDP areas were 52 percent less likely to choose institutional delivery compared to those in LDP areas.

The likelihood of choosing institutional delivery decreased with increased years of schooling of the spouse (OR: 1.16; *p-value ≤ 0*.*01*). Women whose spouses were occupied in business (OR: 2.79; *p-value ≤ 0*.*05*) were 179 percent more likely to deliver their child at an institution compared to the women whose spouses were occupied in agriculture.

Most need factors had a significant (*p-value* ≤ 0.05) positive associations with institutional delivery. Mothers who had complications in earlier pregnancies and also during the last pregnancy period were respectively 287 and 367 percent more likely to deliver their child to a health facility. In addition, mothers who received recommended (≥ 4) ANC than no ANC visits, and sought ANC from graduate doctors compared to their counterparts were 208 and 249 percent more likely to choose institutional delivery respectively.

## Discussions and conclusions

This study fills the gaps regarding the effect of climate change risks in broader contexts (flood, cyclone, and riverbank erosion) in utilizing ANC visits and institutional delivery by comparing HDP and LDP areas. HDP areas are expected to have lower utilization of these two outcome variables of maternal care. The result of this study is aligned with our expectation for institutional delivery but not for ANC use.

This study reveals that the overall utilization of WHO-recommended four or more ANC visits was very low (17%) compared to other studies, which found 26% to 32% by analyzing contemporary national-level data [6, 19, 22, 58]. Like these national level studies, a small-scale study in rural areas also shows similar (31%) evidence [59]. Our result is comparable to the Ethiopian studies conducted in rural areas (15%) [14, 16], where some developing countries had higher utilization of recommended ANC, such as 47% in Sudan, 69% in Nepal, and 80% in China [12, 13, 15]. Consistent with other studies [6, 11, 15, 17, 20], the important factors for the recommended ANC use are the mother’s education, household size and proximity to health center.

The utilization of recommended ANC visits is somewhat higher in HDP areas compared to LDP areas, that was beyond our expectations. However, the difference is not significant at any conventional significance level. As reported in the literature [76] for ANC in HDP areas, more concentration of NGO health service provision may be an underlying factor. Similar evidence was also found in a previous study comparing the recommended number of ANC utilization between flood-affected and non-flood-affected areas [58].

The overall institutional delivery in the rural areas of Bangladesh (21%), as found in our study, is similar to the evidence found in some national level studies [6, 37, 38, 51, 77] except one [10]. However, this is substantially low compared to the developing countries as found in some contemporary studies, such as Haiti (45%), Myanmar (61%), Nepal (66%), Pakistan (53%), India (46%) and Ethiopia (31%) [11, 26, 29, 31, 32, 35].

The multivariate analysis shows that, as expected, women of HDP areas were significantly less likely to choose institutional delivery compared to those of LDP areas, although the difference between HDP (15%) and LDP (20%) areas is not significant for institutional delivery. A small number of observations of institutional delivery in the cells may be accountable for this insignificant result. Note that home delivery was significantly higher in HDP areas compared to LDP areas. A similar level (16%) of the utilization of institutional delivery was also found in a recent study conducted in flood-affected displacement-prone areas [60]. The study found significantly lower institutional delivery among the displaced households (16%) than non-displaced households (60%). Another study based on BDHS 2014 found about 38 percent institutional delivery in flood-affected and non-flood affected areas [58].

Quite expectedly, however, the attendance of physicians such as MBBS doctors, gynecologist, or surgeons at childbirth was significantly lower in HDP areas compared to LDP areas. This may be caused due to significantly lower utilization of private health facilities in HDP areas compared to LDP areas. As reflected in the result, institutional delivery leads to alarmingly more C-section delivery.

The lower utilization of institutional delivery care in HDP areas is reflected by the poor socioeconomic attributes of the respondents. For instance, there is a strong influence of years of schooling of the spouse of pregnant women, which was less in HDP areas compared to LDP areas, on the choice of institutional delivery. Moreover, the spouse’s engagement in business also strongly affected institutional delivery. However, the spouses of HDP areas were less occupied in business and more occupied in agriculture compared to LDP areas. Thus, the low level of awareness and lack of affordability resulting from fragile socio-economic background of the inhabitants of HDP areas affect the utilization of institutional delivery. The poor socio-economic background of the residents of disaster-prone areas was also found accountable for the low utilization of institutional delivery in the literature [60]. Longer distance to the nearest healthcare facility was found to be a prominent factor for overall low utilization of institutional delivery. A significantly longer distance to the nearest healthcare facility may also be a reason behind the low utilization of institutional delivery in HDP areas. In addition, the lack of proper communication and road transportation network in HDP areas hinders pregnant women from visiting long distanced facilities.

The utilization of ANC by graduate physicians leads to increase the level of institutional delivery [11, 32]. Low utilization of institutional delivery in HDP areas may be a telltale sign of considerably inadequate visits to these providers. No significant difference exists in utilization of recommended four or more ANC between HDP and LDP areas. On the other hand, intuitional delivery, especially from graduate physicians, is significantly lower in HDP than in LDP areas. However, the overall utilization of both recommended ANC and institutional delivery is still very low despite the various efforts (e.g., strengthening primary healthcare through establishing community clinics, expanding *Upazila* Health Complex, etc.) undertaken by the government and non-government organizations over time. This is mainly because of the inadequacy of qualified providers and the lack of adequate diagnostic facilities as well as equipment for emergency C-section delivery in *Upazila* and below-level healthcare facilities. Absenteeism and ‘not filling up’ the vacant posts are two major factors for the unavailability of qualified providers in rural areas [78]. These problems are more acute in HDP areas than in LDP areas.

Demand-side factors such as low education and income and employment in agriculture are also accountable for underutilizing ANC and institutional delivery in HDP areas. The lack of affordability to spend on maternal healthcare in private facilities in HDP areas maintain that financial burden may play a telling role in impeding opting for institutional delivery. Significantly lower out-of-pocket payments for maternal healthcare (both ANC and delivery) in HDP areas compared to LDP areas bolster this claim.

The findings suggest devoting more efforts to increasing institutional delivery, especially in HDP areas. Adopting demand-side interventions such as awareness raising and covering more mothers under the maternal allowance program of the Ministry of Women and Children Affairs, which offers a monthly allowance of BDT800 to poor pregnant women, are essential for creating demand as well as making affordable utilization of recommend ANC and institutional delivery. Expanding the maternal voucher scheme of the Ministry of Health and Family Welfare implemented for improving the utilization of maternal healthcare by the poor pregnant mother can also be another demand-side intervention. The local NGOs can be encouraged through Palli Karma-Sohayak Foundation (PKSF) to create more income-generating activities especially off-farm ones. Employment in off-farm activities along with farming will make the households better cope with agricultural production lost due to frequent natural disasters as evident in the literature [79].

Focus also needs to be given to some supply-side interventions, such as general preparedness of the government facilities in the upazila (sub-district) level and below through filling up the vacant post of both technical and support staff and reducing absenteeism by introducing appropriate incentive mechanisms such as remote allowances, better accommodation at a cheaper cost and aligning the higher education opportunity with serving in the disaster-prone areas. Although these are quite unexpected to happen in the short run, efforts need to be continued to make it doable in the long-term. As a short-term solution, the local traditional birth attendants can be trained for better handling home delivery. Some area-specific measures also can be undertaken such as the deployment of boat-based medical teams in coastal and *char* areas. In addition, the ANC and delivery care can be made available in the government facilities of the HDP areas through meeting the staff shortage by employing them on the contractual basis with pay for performance incentives. Public-private partnerships modality can also be introduced.

Thus, HDP areas attack more policy attention to adopt the abovementioned measures for addressing both demand-side and supply-side constraints of the utilization of recommended ANC from graduate physicians and institutional delivery to reduce maternal mortality.

## Limitations

We could not run a separate regression model to identify the influencing factors of ANC and delivery care for LDP and HDP areas due to a lack of adequate observations as extracting the data from a multi-purpose survey. Another limitation is the use of perceived disaster-related data collected from the key informants through a village survey due to the lack of meteorological data in the micro-settings. Although social desirability bias is a common phenomenon in survey-based research, the problem is not acute in our case as we dealt with retrospective events. Moreover, this study used data from a multi-purpose survey which did not include all the individual, social or system-level factors (e.g., individual motivation, self-efficacy, health literacy, traditional health beliefs, media access, and social support).

## Supporting information

S1 Table The unadjusted multinomial logistic model estimation to assess the factors of ANC contacts. (DOCX)

S2 Table The unadjusted logistic model estimation to assess the factors of choice of child delivery place. (DOCX)
