# Supplementary material for: Maternal healthcare utilization in rural Bangladesh: A comparative analysis between high and low disaster-prone areas
Source: PLOS Glob Public Health. 2023 Jul 31;3(7):e0001409. doi: 10.1371/journal.pgph.0001409 (PMC10389743; doi:10.1371/journal.pgph.0001409)
Supplement: S1 Table — (DOCX) [file pgph.0001409.s001.docx]

**S1 Table** The Unadjusted Multinomial Logistic Model Estimation to assess the factors of ANC contacts.

| *Dependent variable:* Number of ANC received (None (0) is the reference category) | | |
| --- | --- | --- |
| Explanatory variables | **Unadjusted model** | |
|  | 1-3 visits | $\geq4$ visits |
| **Predisposing factors** | RRR  (CI) | RRR  (CI) |
| Parity |  |  |
| First | Ref | Ref |
| Second or third | 0.98  (.61 to 1.58) | ${1.48}^{§}$  (.78 to 2.78) |
| Four or more | 0.60*  (.33 to 1.09) | 0.18***  (.05 to .61) |
| Mother’s age (in years) | 0.98  (.94 to 1.02 | 0.97  (.92 to 1.03) |
| Mother’s Education (in years) | 1.15***  (1.07 to 1.23) | 1.31***  (1.18 to 1.45) |
| Religion (1= Muslim) | 0.14*  (.02 to 1.14) | 0.13*  (.01 to 1.14) |
| Spouse’s Education (in years) | 1.16***  (1.09 to 1.25) | 1.22***  (1.13 to 1.33) |
| Number of households had member with at least ten years of education | 1.08  (.62 to 1.86) | 0.84  (.40 to 1.77) |
| Household size | 0.88***  (.79 to .97) | 0.84**  (.72 to .97) |
| Number of family planning discussion session with spouse (1=Multiple times, 0=otherwise) | 2.16***  (1.33 to 3.50) | 3.07***  (1.57 to 6.02) |
| **Enabling Factors** |  |  |
| Occupation of husband | RRR  (CI) | RRR  (CI) |
| Agricultural sector dummy | Ref | Ref |
| Transport sector dummy | 0.93  (.43 to 2.02) | 0.77  (.26 to 2.28) |
| Business sector dummy | 0.89  (.46 to 1.69 | 1.37  (.62 to 3.0) |
| Service sector dummy | 2.57**  (1.13 to 5.86) | 2.62*  (.97 to 7.05) |
| Other dummy | 1.53  (.67 to 3.49) | 1.78  (.65 to 4.88) |
| Per day per capita household consumption (log value) | 2.37***  (1.33 to 4.20) | 3.11***  (1.54 to 6.27) |
|  | 1-3 | $\geq4$ |
|  | RRR  (CI) | RRR  (CI) |
| Having any health shock in the household in last two years (1=yes) | 0.77  (.47 to 1.26) | 1.08  (.57 to 2.04) |
| Total number of illness episodes for all members of the household | 1.03  (.86 to 1.24) | ${1.17}^{§}$  (.93 to 1.47) |
| **External Environmental factors** |  |  |
| Proximity to health facility (in mile) | 0.79***  (.67 to .94) | 0.64***  (.48 to .86) |
|  |  |  |
| Type of disaster-prone areas (1= HDP areas) | 0.62**  (.38 to 1.00) | 1.08  (.58 to 2.00) |
| **Need Factors** |  |  |
| Complicated earlier delivery (1=yes) | ${1.83}^{§}$  (.78 to 4.27) | ${2.02}^{§}$  (.72 to 5.68) |
| Complicated last delivery (1=yes) | 2.14**  (1.11 to 4.15) | ${1.96}^{§}$  (.85 to 4.48) |
| Number of observations | 343 | |
| *Note*s: ***, **, *, § and ¶ indicates significance at 1%, 5%, 10%, 15%, 25% level, respectively. Ref=Reference Category; RRR=Relative Risk Ratio, CI = confidence interval, ANC = antenatal care. | | |
