# Supplementary material for: Maternal healthcare utilization in rural Bangladesh: A comparative analysis between high and low disaster-prone areas
Source: PLOS Glob Public Health. 2023 Jul 31;3(7):e0001409. doi: 10.1371/journal.pgph.0001409 (PMC10389743; doi:10.1371/journal.pgph.0001409)
Supplement: S2 Table — (DOCX) [file pgph.0001409.s002.docx]

**S2 Table** The unadjusted Logistic model estimation to assess the factors of choice of child delivery place

| *Dependent variable: choice of child delivery place (1=at institution; 0=at home)* | |
| --- | --- |
| Explanatory variables | OR  (CI) |
| **Predisposing factors** |  |
| Parity |  |
| First | Ref |
| Second or third | 0.86  (.51 to 1.45) |
| Fourth or more | 0.12***  (.03 to .50) |
| Mother’s age at birth (in years) | 0.93***  (.88 to 98) |
| Mother’s Education (in years) | 1.26***  (1.16 to 1.38) |
| Religion (1= Muslim) | ${0.51}^{§}$  (.17 to 1.54) |
| Spouse’s Education (in years) | 1.22***  (1.14 to 1.31) |
| Number of households had members with at least ten years of education (1=yes) | 1.21  (.67 to 2.17) |
| Household size | 0.95  (.85 to 1.07) |
| Decision maker of choosing child/s birthplace (1= Husband-Wife together; 0=Others) | ${.70}^{¶}$  (.41 to 1.18) |
| Number of family planning discussion sessions with spouse (1=Multiple times, 0=otherwise) | 2.25***  (1.27 to 4.01 |
| **Enabling Factors** |  |
| Occupation of spouse |  |
| Agricultural sector dummy | Ref |
| Transport sector dummy | 0.98  (.41 to 2.34) |
| Business sector dummy | 1.74*  (.92 to 3.29) |
| Service sector dummy | 2.59***  (1.33 to 5.04) |
| Other dummy | ${0.43}^{§}$  (.15 to 1.24) |
| Per day per capita household consumption (log value) | 2.53***  (1.48 to 4.31) |
| Having any health shock in the household in the last two years (1=yes) | ${0.64}^{§}$  (.36 to 1.12) |
| Total number of illness episodes for all members of the household | ${0.88}^{¶}$  (.71 to 1.08) |
| **External Environmental Factors** |  |
| Proximity to health facility (in miles) | 0.67***  (.51 to .88) |
| Type of disaster-prone areas (1= HDP areas) | 0.52*  (.30 to .89) |
| **Need Factors** |  |
| Complicated earlier delivery (1=yes) | 2.29**  (1.10 to 4.75) |
| Complicated last delivery (1=yes) | 3.82***  (2.13 to 6.84) |
| The number of ANC received |  |
| None | Ref |
| Inadequate (1-3 visits) | 1.66*  (.98 to 2.81) |
| Recommended (4 or more visits) | 2.99***  (1.64 to 5.48) |
| ANC sought from graduate doctors (1=Yes) | 5.62***  (3.21 to 9.86) |
| No. of TT received |  |
| None |  |
| Inadequate (1-4) | 1.66*  (.90 to 3.07) |
| Adequate (5) | 2.02*  (.87 to 4.72) |
| Number of observations | 343 |
| *Note*s: ***, **, *, § and ¶ indicates significance at 1%, 5%, 10%, 15%, 25% level, respectively. Ref=Reference Category; OR=Odds Ratio, CI = confidence interval, ANC = antenatal care | |
